# Food supplements to reduce stunting in Pakistan: a process evaluation of community dynamics shaping uptake

**DOI:** 10.1186/s12889-020-09103-8

**Published:** 2020-07-02

**Authors:** Shehla Zaidi, Jai K. Das, Gul Nawaz Khan, Rabia Najmi, Mashal Murad Shah, Sajid B. Soofi

**Affiliations:** 1 Department of Community Health Sciences, Aga Khan University, Karachi, Pakistan; 2 Division of Women and Child Health, Aga Khan University, Karachi, Pakistan; 3 Department of Pediatrics and Child Health, Aga Khan University, Karachi, Pakistan

**Keywords:** Stunting, Food supplements, Usage, Acceptability, Community health workers

## Abstract

**Background:**

There is an increasing interest in use of food supplements to prevent childhood stunting, however the evidence on the process indicators is scarce. We in this study explore the barriers to the effective implementation of food supplementation programs and the possible mitigation strategies which can guide the design of future programs.

**Methods:**

We undertook a process evaluation of a stunting prevention food supplementation pilot program in rural Pakistan that distributed Wheat Soy Blend (WSB) to pregnant & lactating women, and Lipid-based Nutrient Supplement (LNS) and micronutrient powder (MNP) to < 5 years children. We used a mixed methods approach through a quantitative survey of 800 households and conducted 18 focused group discussion (FGDs) (with male and female caregivers), 4 FGDs (with Community Health Workers (CHWs)) and 22 key informant interviews (with district stakeholders) to evaluate the community side factors affecting uptake through five parameters: value, acceptability, receipt of supplement, usage and correct dosage.

**Results:**

The findings show that proportionately few beneficiaries consumed the full dose of supplements, despite reasonable knowledge amongst caregivers. Sharing of supplements with other household member was common, and the full monthly stock was usually not received. Qualitative findings suggest that caregivers did not associate food supplements with stunting prevention. WSB was well accepted as an extra ration, LNS was popular due its chocolaty taste and texture, whereas MNP sprinkles were perceived to be of little value. The cultural food practices led to common sharing, whereas interaction with CHWs was minimal for nutrition counselling. Qualitative findings also indicate CHWs related programmatic constraints of low motivation, multi-tasking, inadequate counselling skills and weak supervision.

**Conclusion:**

We conclude that the community acceptability of food supplements does not translate into optimal consumption. Hence a greater emphasis is needed on context specific demand creation and focusing on the supply side constraints with improved logistical planning, enhanced motivation and supervision of community workers with involvement of multiple stakeholders. While, similar studies are needed in varying contexts to help frame universal guidelines.

**Trial registration:**

ClinicalTrials.gov Identifier: NCT02422953. Registered on April 22, 2015.

## Background

The burden of undernutrition remains unacceptably high with 800 million people globally and 780 million of these residing in low-to-middle income countries, especially in Sub-Saharan Africa and South Asia [1]. Children under the age of five years are highly vulnerable, as 150.8 million children are stunted and 50.5 million are wasted [2]. Apart from other factors, inadequate food intake and poor dietary quality are responsible directly or indirectly for causing ill-health with six of the top eleven global risk factors being associated with dietary imbalances [1] and in 2017, 11 million deaths and 255 million disability-adjusted life years (DALYs) were attributable to dietary risk factors [3].

The Provision of packaged specialised food supplements are argued to be a rapid and low cost approach to prevent under-nutrition in vulnerable groups such as pregnant-lactating women (PLW) and young children 6–59 months of age [4, 5] and more specifically focusing on the critical 1000 day period from conception to 2 years of age [6, 7]. These specialized packaged foods comprise of various products which are carefully designed to cater the needs of specific vulnerable groups. Fortified Blended Foods (FBFs) are designed for PLW and consist of a blend of partially precooked cereals in either wheat or corn base and fortified with vitamins and minerals which is mixed with water and cooked [8]. Ready to Use Foods (RUF) are designed for children 6–59 months of age and are eaten in small quantities as a supplement to regular diet and are prepared in a lipid base providing proteins, fats and micronutrients [9, 10]. Micronutrient powders (MNP) are single use powder packets of vitamins and minerals which is sprinkled on cooked meals of children [11]. High energy biscuits comprising of proteins and minerals have also been designed for children and adults especially in emergency settings. These food supplements are usually provided for the prevention of stunting through community-based approaches to ensure access to the most marginalized with appropriate counselling and support [12].

Food supplementation programs for the prevention of stunting have been implemented as part of food security measures in many countries including India, Malawi, Bangladesh, Madagascar and Ghana [13–16]. The existing evidence of the impact of FBF and RUFs to prevent stunting remains inconclusive [4, 11, 14, 17, 18]. Moreover, most of the available evidence is from trials, where there is stringent monitoring for food distribution and greater support to mothers [14, 15, 18, 19]. However, in large scale health programs, the distribution and effective uptake of food supplements is certain to be more challenging especially if the food is to be taken regularly over a longer period of time.

There is an increasing body of evidence for the impact of ready-to-use therapeutic foods (RUTFs) for under-nutrition, but relatively less evidence on the effect of food supplements for the prevention of stunting. Mixed method studies have also been conducted to explore the adherence and acceptability of food supplements within the community and to understand the contextual factors associated with appropriate consumption [20]. The available evidence on the factors influencing the usage of food supplementation is presently small and emerging, and there is a greater need for evidence emerging from real programmatic settings and outside of controlled trial settings as these could provide real insights on how community adherence can be improved.

We present here the findings from a process evaluation of a pilot study on food supplement based stunting prevention program conducted in two rural underserved districts of Sindh province in Pakistan. This paper focuses on the process evaluation, while a separate paper would be published on the effectiveness of the intervention. We used a mixed methods approach to delve into community dynamics around the usage of food supplements to prevent stunting, hence contributing to the empirical insights for effective community uptake of food supplements when designing food supplementation programs in food insecure settings. This study was conducted in Pakistan 40.2% children are stunted, the highest in South Asia and nearly twice as much as the global prevalence [21].

## Method

### Setting

The is a process evaluation of a two-year stunting prevention project (2014–2016), where food supplements were provided to PLW and children 6–59 months of age in two districts (Thatta and Sujawal) of Pakistan with an established high prevalence of childhood stunting. The food Supplements were provided in the 29 Union Councils (UCs) -the lowest administrative unit- of the total of 55 UCs of the two districts through community-based Lady Health Workers (LHWs). The food supplements consisted of locally produced LNS (Wawamum – chocolate drink) given to children from 6 to 23 months of age, MNPs (powder to be sprinkled on food) to children 24–59 months of age and Wheat Soy Blend (WSB) (chickpea based fortified food to be mixed with flour) to PLW during pregnancy and for 6 months after giving birth (Table 1). Besides the provision of food supplements, counselling was provided on food supplement use and infant and young child feeding (IYCF) (early initiation and exclusive breastfeeding, sustained breastfeeding, complementary feeding and hygiene practices). The details of the program and methodology are provided elsewhere [22].

**Table 1:** Food Supplements and targeted beneficiaries

Supplement	Target group	Dosage/ Frequency	Consumption
LNS (Wawa Mum)	Children aged 6–23 months	1 sachet (50 g) Once a day	Directly from the sachet
MNP	Children aged 24–59 months	1 sachet Every other day	Sprinkled over semi-solid or solid food
WSB	Pregnant and/or lactating women	5 kg (2 bags) Spread throughout the month	Making bread or desserts

*LNS* Lipid based nutrient supplement; *MNP* Micronutrient powder; *WSB* Wheat Soy Blend

The LHW program has been running for several years in Pakistan and operates through salaried village-based lady health workers who provide frontline maternal and childcare preventive services growth monitoring, and identify severe disease and ensure timely referrals. Each LHW has a health house in her village as the centre point for health awareness sessions, and also conducts home visits on a monthly basis. The LHWs are supervised by Lady Health Supervisors (LHS) based at a health facility where they also collect commodities and submit monthly reports.

### Food intervention

In this program**,** all the PLW and children < 5 years of age residing in the 29 LHW covered UCs were the beneficiaries of the food supplementation program irrespective of their nutritional status. Food stocks (LNS for children aged 6–23 months, MNP for children aged 24–59 months and WSB for PLW) were collected by LHWs during their monthly visit to the health centre and mothers then visited the LHW health house in their respective villages to receive the monthly supplements. The mothers were provided information on food supplement use and further follow-up on food usage were done by LHWs during routine household visits.

### Process evaluation

In this study, we investigated the community dynamics related to the uptake of ready to use food supplements by the intended recipients through a cross-sectional mixed methods assessment. This study was based on the five key parameters critical to the success of a food supplementation program which were identified through a review of the existing literature and comprised of

- Value: recognising the value or importance of food supplements for health and disease.
- Acceptability: Acceptability typically expressed in terms of likes or dislikes of packaging, texture, taste and smell [23].
- Receipt of supplements: Delivery actions for supply and distribution of food supplements require attention to effectively integrate food supplementation into infant and young child feeding practices [12]
- Targeting compliance: Supplements are created for specific target populations, and it is important for only those populations consume the supplements in adequate amounts [10].
- Dosage compliance: Compliance with adequate dosage and frequency is required over long stretches of time, requiring effective filtration of information to beneficiaries [24].

### Data tools

We used a range of mixed methods to explore the above parameters (Table 2):

i) Household quantitative survey of PLW and mothers of children under 5 years of age- to examine receipt of food supplies, knowledge and consumption.
ii) Focused group discussions (FGDs) with female and male caregivers probing value, acceptability, receipt of supplies, consumption by targeted beneficiaries
iii) FGDs with LHWs probing community uptake and delivery factors affecting usage
iv) Key informant interviews (KIIs) with district stakeholders probing community uptake and delivery factors affecting usage

**Table 2:** The data collection tools and parameters

Study area	Parameters	Tools (***N***)	Study Participants	Themes Explored
Community level dynamics affecting uptake of food supplements by targeted beneficiaries	-Are food supplements being received by households? -Does the community understand the value and purpose of supplements? -Is there acceptability of food supplements amongst target groups and particular likes and dislikes associated with different supplements? Is there consumption by required beneficiaries and in sufficient quantities	HH Survey (*n* = 806)	Mothers	-Receipt of food supplements -Usage of food supplements by target groups -Knowledge of food supplement usage
		Community perceptions FGDs (*n* = 18)	PLW: 2 FGD Fathers & male HH members of < 5 children: 6 FGDs	-Value of food supplements and willingness to pay -Acceptability of food supplements - Receipt of food supplements -Usage of food supplements
		Health provider feedback FGDs (*n* = 4) KIIs (*n* = 22)	FGDs: LHWs KIs: district and union council stakeholders	Enablers & Barriers related to: -Uptake of food supplements by community -Delivery of food supplements

*FGD* Focused Group Discussion; *HH* Household; *KII* Key Informant Interviews; *LHW* Lady Health Worker; *PLW* Pregnant Lactating Women

### Data collection

#### Household survey

A structured paper-based questionnaire was used to collect data from randomly identified households (HH). Sample size was calculated using the comparison of sequential surveys approach to detect a 15% difference in ever receiving food supplements by eligible population. Sample size calculations considered the percentage of food supplement received during the previous process assessment i.e. 69% and design effect of two and the total sample size calculated was 806 to detect a 15% difference with 80% power and 5% level of significance. No minors (16>) were included in the sample. The HH survey was conducted in 12 UCs randomly selected from both districts and the probability proportional to size (PPS) sampling method was used to randomly select houses from the lists of HH already available with the LHWs (Additional file 1).

#### FGDs with caregivers

We randomly selected 12 UCs from the targeted 29 UCs to conduct FGDs. A total of 18 FGDs were conducted with community members: twelve FGDs were conducted with mothers and mothers-in-law, and six with male members of households. Each FGD had 8–10 participants, and lasted around 2 h. A topic guide with probes was used to gather information on value, distribution, acceptability, and usage of food supplements during these discussions (Additional file 2).

#### FGDs with LHWs

*A total of 4 FGDs were conducted with LHWs which were randomly selected from 12 UCs. Each FGD had 8–10 participants and lasted around 2 h. A topic guide with probes was used to probe LHW perceptions on community uptake of supplements, underlying factors and LHW experiences on delivery of food supplements (Additional file*2*).*

#### KIs with district stakeholders

A total of 22 KIIs were carried out with district stakeholders, including the district health office staff, union council representatives, community-based organizations, and Lady Health Supervisors (LHS) overseeing LHWs. A topic guide was used to probe perceptions on community uptake of supplements, factors underlying community uptake and perceptions of programmatic delivery of food supplements (Additional file 3)***.***

### Quality assurance

Two field-based teams were formed; one for household survey and the other for qualitative investigation and each was headed by a field supervisor. The study lead and research specialist trained both the team on study tools, oversaw data collection and real time analysis. Household questionnaire was pre-tested before data collection, validation checks were done on 5% of the forms within the same day and errors corrected. Data forms after being checked for completeness by field supervisor were entered into SPSS using double entry to minimise errors.

FGDs with community members were conducted in an accessible location, usually in a village home or school chosen by the participants. Space at local government health facilities was used for FGDs with LHWs as it is a common convening point. Each FGD was conducted by a pair of facilitators and note taker, held in local Sindhi language, and principles of free flow of conversation were established at the outset. Tape recorders were used with permission of the participants. FGDs and KII were conducted in local language, transcription was carried out during field data collection, and after checking with the audio recordings were translated to English.

### Data analysis

Quantitative data was analyzed in Stata version 14. We performed descriptive analysis and frequencies were generated for all of the categorical variables. The transcripts from FGDs and KIIs were manually analyzed using inductive thematic analysis and content coded a priori in line with the identified five parameters. Coding was reviewed by the study team, discrepancies were discussed, relationship between themes was discussed and new codes created where felt necessary. Triangulation of emerging findings was done across HH survey, community FGDs, LHW FGDs and KIs to identify commonalities and differences.

### Ethical considerations

The project obtained ethical approval from the Ethics Review Committee (ERC) of Aga Khan University (GN: 2919-Ped-ERC-14) and the National Bioethics Committee (NBC) (4–87/14/NBC-147/RDC/624) of the Pakistan Medical and Research Council. Written informed consent was obtained from survey participants prior to collection of data. The FGDs and KIs began with introduction of the study, re-confirmation of interest to participate in the discussion, and maintained confidentiality of respondents’ information. Identifying information of respondents was removed in transcription, analysis and reporting, substituting with numeric codes. All computerised data was encrypted, and hard copies were stored in locked cabinets to ensure confidentiality of data.

## Results

### Household survey

Out of the 806 households surveyed, there were 203 HHs with children aged 6–23 months, 200 HHs had children aged 24–59 months and 403 HHs with PLW. Most of the mothers (99.6%) in the catchment area were aware of the collection points for the food supplements, 78% received food supplements during the previous month, whereas 22% of the HHs did not receive food supplements in the previous month. The major reasons for not receiving the food supplement were that the mothers either forgot to collect (10%) or were busy in household work (5%) or the mothers visited the health house but the food supplements were not available (5%) (Table 3).

**Table 3:** Findings from the quantitative cross-sectional survey

**Total**	***N (%)*** **806 (100)**
Proportion of mothers aware of delivery points	803 (99.6)
Any food supplements received in the previous month	626 (77.7)
Not receiving food supplements	180 (22.3)
*Forgot to receive/visit LHW’s health house*	83 (10.3)
*Busy in household work*	44 (5.4)
*Visited but supplements not available*	39 (4.8)
*Visited but LHW was absent*	6 (0.7)
*Other*	8 (1.0)
**Total**	***N (%)*** **806 (100)**
Proportion of mothers aware of delivery points	803 (99.6)
Any food supplements received in the previous month	626 (77.7)
Not receiving food supplements	180 (22.3)
*Forgot to receive/visit LHW’s health house*	83 (10.3)
*Busy in household work*	44 (5.4)
*Visited but supplements not available*	39 (4.8)
*Visited but LHW was absent*	6 (0.7)
*Other*	8 (1.0)

*LHW* Lady Health Worker

However, everyone did not receive the full stock of supplements; only 50% of households received full stock for LNS, 61% for MNP, 63% for WSB at the time of survey (Table 4). The supplements were often shared with other members of the household; LNS were shared in 45% of eligible households, WSB in 34% of households and MNPs in 13% of households. The eligible individuals did not always consume food supplements in the required dose; 20% used the full dose of LNS, 30% took the full does of WSB and 32% took the full dose of MNP. The knowledge of correct use and dose was fairly high, as most mothers (80%) were aware of the correct dose of LNS and WSB and all (100%) were aware of the correct method of consumption for LNS and WSB. Comparatively fewer mothers (64%) knew the correct dosage of MNP although most (80%) knew how to correctly use MNP.

**Table 4:** Knowledge and compliance of care providers for use of food supplements.

Variable	LNS (children aged 6–23 months) ***N (%)-*** 203 (100)	MNP (children aged 24–59 months) ***N (%) –*** 200 (100)	WSB (PLW) ***N (%)-*** 403 (100)
Knowledge of correct dose	165 (81.2)	127 (63.5)	334 (82.8)
Knowledge of correct method of preparation	203 (100)	159 (79.5)	403 (100)
Full monthly quota of supplement received	101 (49.7)	122 (61.0)	253 (62.7)
Partial or no supplement received	102 (50.3)	78 (39.0)	150 (37.3)
Sharing of supplement with other HH members	88 (45.0)	25 (13.0)	129 (33.6)
Use of supplement by eligible individual *Full dose of supplement used* *Partial or no dose of supplement used*	108 (55.0) 42 (20.7) 161 (79.3)	174 (87.0) 63 (31.5) 137 (68.5)	255 (66.4) 121 (30.0) 282 (70.0)

*LNS* Lipid based nutrient supplement; *MNP* Micronutrient powder; *WSB* Wheat Soy Blend.

### FGDs with community

#### Value of food supplements

Most of the caregivers did not perceive stunting as a health concern, as they commonly believe that stunting is a hereditary condition and God has ordained a person’s height. LNS supplements were believed to be associated with greater ‘physical strength’ and ‘energy’ for people of all ages. WSB was regarded by some caregivers to positively impact child’s health at birth, while others regarded this as an extra bag of ration. Most of the caregivers were willing to use supplements only if were provided free of cost, while only a few were willing to pay a token amount for these food supplements, with a higher willingness to pay (WTP) for WSB and lowest for MNP. WTP reported by participants was up to forty rupees for WSB, ten rupees for LNS and maximum three rupees for MNP for a single pack of the supplement. Caregivers reported that they would not be interested in purchasing food supplements and believed it would be more beneficial to spend their money on other rations such as cooking oil and wheat, which could feed the entire family instead of select groups. Participants stated that they would not mind using WSB and LNS if provided free of cost. .

*“It’s [Stunting] natural for children because it depends on parents’ heights. If parents are smaller, their children will also be small” (LM)*

*“It [WSB] is good thing and gives physical strength [energy] to the pregnant and lactating women”*

#### Receipt of food supplements

Most mothers knew that the LHWs health houses were the collection point for the food supplements and where they were located. However, mothers reported that they did not always got the full stock of supplies. Sometimes they were unable to pick up the supplements due to household chores and instead sent their children or spouse to pick up the supplies. However, husbands and children often did not interact with the LHWs, and did not stop to listen to the LHWs’ instructions on its use and benefits. Mothers expressed that LHWs distributed supplies in a hurry and there was little time to understand why the supplements should be used. They also mentioned that although the LHWs did make household visits, but growth monitoring of child was rarely carried out and there was little discussion on food supplement use.

*“ …, I don’t get the food supplement every time, as she [LHW] says stock has finished and asks us to wait for a few days.” (LM)*

*“We should sit together with her [LHW] to discuss about the use and benefits of the supplements and other issues.” (PW)*

#### Acceptability of food supplements

Information about the acceptability of the food supplements was probed based on their colour, texture, odour, and packaging. Participants expressed strong liking for the taste and texture of WSB and believed that all family members were entitled to consume it, although they were indifferent about its colour, odour and packaging. The preferred use of WSB was through mixing with wheat flour to make chappatis served at meals. Participants enthused over the taste of LNS due to its “chocolate” flavour and syrupy liquid texture. Caregivers were largely hesitant to use MNP and also reported that children did not adequately take it. It was believed that the powder changed the colour and taste of the food that it was sprinkled over, hence barring children from consuming their meals. Some caregivers also expressed that children experienced bouts of diarrhoea after consuming MNPs.

*“…yes because its taste is good, so all children have it and they are happy to have Wawa Mum” (PW)*

*“All members have it [WSB when it is prepared and mixed in flour [aata] and meal [mani] is made*

*“WSB tastes good when prepared and mixed with anything; people like it.” (LM)*

*“Wawa Mum (LNS) is good for children’s health. It gives them energy.” (LM)*

*“The taste of [MNP] is not liked by children. It changes color of certain food products.” (Grand-mother)*

#### Usage of food supplements

Sharing of WSB and LNS was commonly reported among the household members. WSB was usually served to all household members mixed in chappatis (bread) for daily meals. Caregivers felt that it was culturally unacceptable for mothers to consume separate chappatis made of WSB flour and it was also considered as an extra work to make two doughs and chappatis (bread). LNS was reported to be popular amongst children and adults due to its taste and this led to consumption by older siblings and sometimes even by adults. Most caregivers felt that it would be unfair to restrain older children from having a chocolate treat. MNP was not shared due to perceived unpleasant taste and issue of food changing colour when sprinkled with MNP.

### FGDs with LHWs

#### Community uptake

LHWs reported that households were always willing to receive extra food parcels, however expressed concern that food sharing was common and hence the target beneficiaries did not always receive the adequate amount of the food supplements. LHWs believed that village-level committees comprised of influential and educated people from the community would be helpful in ensuring that the right beneficiaries consume food supplements.

#### Food supplement delivery

LHWs were concerned that substantial time was required in maintaining records of supplements and counselling of households on supplement use and they did not get sufficient time from their usual chores to attend to these tasks. They also felt demoralised that their tasks kept on increasing and there was little support available from their supervisors. LHWs also mentioned delays in re-stocking of supplements which they attributed to be a major reason for households not being able to obtain full stock of supplements. According to LHWs, their transport allowance was meagre to regularly fetch supplies while on the other hand there was rising demand from the community for WSB and LNS.

*“We don’t get salary and food distribution incentive on time.” (LHW)*

*“At village level there should be committees of influential people who can look after the proper usage and also help us in distribution, otherwise people will continue to demand food supplements for the ineligible ones” (LHW)*

### KIIs with district stakeholders

#### Community uptake of food supplements

KIs expressed concerns that the community is poorly aware of the importance of food supplements and that the critical step prior to food distribution should have been to sufficiently sensitize the community on why to use and who should use. KIs reported that certain segments of the community believed that these supplements are supplied by foreign agencies with malicious intent, hence awareness is all the more required to dispel these suspicions. KIs largely felt that the community does not get sufficient interaction with LHWs, with interaction being mostly confined to polio days, and this was reported as another hindrance to adequate uptake of food supplements. They believed that LHWs should be re-established as primary care givers with the community, particularly with women. This would then also help with supplement use for children and mothers.

#### Delivery of food supplements

There was also concern amongst KIs on record keeping of distribution and usage of food supplements, and many expressed their desire for regular review of such records. LHSs blamed low literacy of LHWs for weak record keeping, yet others believed that record keeping has less to do with competency and more with insufficient emphasis to monitoring. While all key informants recognised that monetary support is required for regular delivery of supplements to health worker houses, there was less consensus on why monetary support is not delivering results so far. LHSs were to blame for slow release of transport allowance to LHWs, whereas the district health office distributing food supplements stated that the transport allowance were often withheld until monitoring reports were submitted by health workers. Stakeholders doubted that LHWs were adequately trained to deliver awareness to the community and stated that community awareness requires considerable support.

*“There should be trust of people on her [LHW] but it is only possible when she performs her duties honestly, she only comes for polio drops, and doesn’t come otherwise.”*

*“There are some religious leaders who misguide people and say that it is western wrold tactics to provide these supplements to us which are mixed up with some [haraam] prohibited food/things.”*

*“There is also issue that a large number of beneficiaries don’t realize that this program is for their good, there is a need of educating the people more on that.”*

*“There are some LHWs who are weak at maintaining records; we need to solve this issue on priority basis.”*

*“Many LHWs don’t submit monthly reports on time but still demand timely provision of incentives and this is not possible that they can get incentive without submission of timely monthly reports.”*

## Discussion

There is an increasing interest in the use of food supplements for the prevention of childhood stunting, however the evidence only from strictly monitored trials remains incomplete, hence meriting the exploration of contextual process indicators. This study contributes to the much needed evidence on the perceptions of the community and the most peripheral health workers and this evidence can entail the factors which would need the most emphasis, so that the large scale food supplementation programs can be successful in reducing childhood stunting.

The findings from this study (Table 5) show that the consumption of full dosage of supplements by intended target groups was sup-optimal for all the three supplements, despite reasonable knowledge amongst care-givers. The quantitative findings also suggest issues with the supply chain, from the supply to the health facilities, lady health workers and to the pickup by the community. WSB was well accepted as an extra ration and LNS was popular due its chocolate taste and texture, whereas MNP sprinkles were perceived to be of little value as they were disliked by children and linked with diarrhea. Sharing was also common amongst the other household members, especially for WSB and LNS. The community largely did not perceive stunting as a problem but considered it due to God’s will and due to the family heredity. While community members were willing to utilise food supplements if provided free of cost there was little WTP for the supplements.

**Table 5:** Summary of key findings

Value of Supplements	Distribution of Supplements	LHWs	Acceptability	Usage of Supplements	Value of Supplements
HH Survey					
	-Supplement not available -majority of mothers were aware of delivery points	-LHW absent at health house			-Target groups did not receive adequate amounts
Community Perceptions: FGDs with Family Members					
-Stunting not viewed as a problem- seen as genetic and God’s will/natural Mothers could see improvement in child’s growth post-supplements	-Information on usage not provided to family members who collected supplements	*-Correct usage technique only taught to mothers by LHWs* *LHWs hurried the sharing of information*	**-WSB-** taste and texture liked by PLW; seen as source of energy and provided physical strength **-LNS-** children liked chocolatey taste **-MNP-** changed the colour of food and taste, so was not liked by target population (or otherwise).	-Lack of trust regarding government intervention -Village elders volunteered to play a positive and productive role in promotion and encourage use of food supplements in their community	- LNS and WSB were shared by household members
Healthcare Provider Feedback: FGDs with LHWs					
	-Supplements not restocked due to transport allowance issues -Male members of the households that come to collect supplements do not wish to stay and learn about usage technique or dosage.	-LHWs felt overwhelmed by multiple tasks -Expressed need for support by LHSs	-Need for village level committee to supervise education and distribution of supplements by elders and educated community members	Demotivating factors affecting usage: -lack of time, supplies, oversight, skills, trainings and support by LHSs	-Concerned regarding target groups not receiving adequate amounts due to sharing of supplements
District Stakeholders Feedback: KIIs					
-Benefits of supplements to alleviate stunting not understood	-Transport allowance is not regularly provided for restocking and transport of supplements	-LHSs stated that LHWs need more education and training on community awareness Poor record keeping by LHWs		-LHWs focused more on anti-polio drives and family planning Multiple commitments make it difficult to effectively run supplement program	

*LHW* Lady Health Worker; *LNS* lipid based nutrient supplement; *MNP***Micronutrient** powder; *WSB* Wheat Soy Blend; *FGD* Focused Group Discussion; *HH* Household; *KII* Key Informant Interviews; *PLW*: Pregnant = Lactating Women

The major issue for the effective utilisation of the intervention was the lack of communication between the community and the LHWs and the major barriers from the LHW side were lack of support and time for these additional activities. There was poor awareness in the community as well as shortfalls in the food supplementation delivery process. The LHWs didn’t have sufficient time due to keeping records and other tasks, were not adequately trained for BCC and were not supervised adequately. There was reluctance from CHWs and field supervisors to absorb food supplementation into their regular routine of work unless supported by extra work stipends, and even then it was felt to overstretch their capacity to deliver. Reliance on CHWs to transport heavy supplies was considered problematic as transportation expense were not timely disbursed. KIIs from district stakeholders also felt the need for more concerted community awareness as well as wider sharing of food monitoring information with public stakeholders. To strengthen the behavior change communication, and to ensure correct information dissemination and consistent monitoring, multiple stakeholders should be mobilized including the LHS,, community midwives (CMWs) and village elders.

The trials conducted in Ghana, Haiti, Peru, Bangladesh and Malawi show wide acceptability of food supplements [13–15, 25, 26]. However, evidence from Ghana and Malawi found low WTP for food supplements in households indicating continued subsidization in provision of food supplements [27]. The sharing of supplements with other household members has been reported in at least two other studies, and highlights cultural imperatives to feed all family members and motherly instinct to share food among all her children that can hinder deliberative targeting of food supplements [13, 14]. Other studies have also found that the distance of the collection point, delay in funds and supplement distribution affect the delivery and use of the supplements [28–30]. There is little evidence on community health workers and their role in supporting food supplement usage, with some evidence indicating inadequate capability of the community workers detrimentally impacting food supplements usage [29, 31].

The strength of our study is that firstly it is not confined to a controlled trial environment in investigation of community uptake of food supplements. Secondly it explores different parameters of uptake drawn from available literature including value, acceptability, receipt of food, usage and targeting compliance. Third, it applies a diverse range of quantitative and qualitative methods to explore food uptake and also distills findings across the levels of community, health workers and district supervisors. Finally, it inductively picks up issues related to community health worker delivery as part of village-based food supplement distribution, an area that has been extensively explored in existing body of literature and provides powerful compelling reasons that need to be acted on to improve food uptake. One of the limitations of this study was not designed to determine diets of children and hence at least the quantitative findings are limited to supplements received and used in the last month, however the qualitative investigation draws on accumulative experience over time. Our study is a descriptive study and does not attempt to test a specific hypothesis and assign quantitative scores. Another limitation was not collecting data on number of children < 5 years in the household, compliance of food supplement and sharing with siblings for all children within household.

Our study shows that blanket food supplementation in communities for certain high-risk groups (children, PLW) is not likely to be seen as targeted to these individuals. Even when there is awareness, the moral obligation and reluctance to refuse results in sharing with other family members.

## Conclusion

We conclude that provision of food supplements to vulnerable populations must be rolled out with caution, with careful attention to village-based dynamics in designing an effective program. Community acceptability of food supplements does not translate into optimal consumption, instead a greater emphasis is needed on demand creation amongst caregivers, countering food sharing practices, implementing efficient logistical mechanisms and moving from sole reliance on community health workers to a broadening of food delivery and behavioural change alternatives. Our study underscores the challenges for community health workers given these additional tasks and a need for additional support with supervision, and finding ways to enhance motivation with incentives. The food supplementation programs in the similar contexts need to meticulously plan and cater to the challenges with context specific planning focused towards improved worker motivation, efficient delivery channels, enhanced communication and involvement of multiple stakeholders. There is a need for similar studies in varying contexts around various low- and middle- income countries which would help form universal guidelines.

## Supplementary information

**Additional file 1: Annex 1** HH Survey Questionnaire House Hold Survey Questionnaire used in the study

**Additional file 2: Annex 2.** FGD Guide Focus Group Discussion guide used in the study

**Additional file 3: Annex 3.** KII Guide Key Informant Interview guide used in the study

## Data Availability

The datasets used and/or analysed during the current study are available from the corresponding author on reasonable request.

## Abbreviations

ERC: Ethical review committee
FGD: Focus group discussion
IYCF: Infant and young child feeding practices
KIIs: Key informant interviews
LHW: Lady health worker
LMICs: Low-income and middle-income countries
LNS: Lipid-based nutrient supplements
MNP: Micronutrient powder
NBC: National bio-ethics committee
PDHS: Pakistan demographic health survey
PLW: Pregnant and lactating women
PPS: Proportion to population size
UCs: Union council
WHO: Word health organization
WSB: Wheat soy blend
WTP: Willingness to pay
