# Supplementary material for: Food supplements to reduce stunting in Pakistan: a process evaluation of community dynamics shaping uptake
Source: BMC Public Health. 2020 Jul 2;20:1046. doi: 10.1186/s12889-020-09103-8 (PMC7331235; doi:10.1186/s12889-020-09103-8)
Supplement: Supplementary file 1 — Additional file 1: Annex 1 HH Survey Questionnaire House Hold Survey Questionnaire used in the study [file 12889_2020_9103_MOESM1_ESM.doc]

**Effectiveness of food based interventions to prevent stunting among children in Thatta & Sajawal Districts, Sindh Province**

**Household Survey Questionnaire for Process Evaluation**

Name of Respondent: _________________________ W/O______________________________________

Name of Village: _____________________________ Name of UC: ________________________________

Age: ________Education: ____________ Occupation: ____________ Date of Interview: _______________

| **Specify the study participant** | | | |
| --- | --- | --- | --- |
| **1 = PW** | **2 = LM** | **3 = Children 6-23m** | **4 = Children 24-59m** |

| **Q.#** | **Questions** | **Responses** | | | **Skip** | | | |
| --- | --- | --- | --- | --- | --- | --- | --- | --- |
| **Section A: Indicators related to receiving of food supplements & usage** | | | | | | | | |
| 1 | Do you know from where to receive the food supplements? | 1 = Yes | 2 = No | | If NO go to Q. 2 | | | |
| 1.1 | If yes, where to receive? |  | | | | | | |
| 2 | Did you receive food supplements during last month? | 1 = Yes | 2 = No | | If yes go to Q 2.1 or 2.2 or 2.3  If No go to Q.4 | | | |
| 2.1 | If yes, how many sachets of Wawamum did you received & used? | Received: _________ Used: _________ | | | | | | 99 = NA |
| 2.2 | If yes, how many sachets of MNP did you received & used? | Received: _________ Used: _________ | | | | | | 99 = NA |
| 2.3 | If yes, how many bags of WSB did you received & used? | Received: _________ Used: _________ | | | | | | 99 = NA |
| 2.4 | If Not received, why (reasons) | 1. Busy in HH work 2. Forget to receive/visit LHW’s HH 3. Visited but supplements not available 4. Visited but LHW was absent   Other Specify …………………………. | | | | | |  |
| 3 | Have you ever been communicated information on whom to feed the supplements & IYCF? | 1 = Yes | | 2 = No | | | | If no go to Q.4 |
| 3.1 | If Yes, who communicated the information to you? | 1. LHW 2. WFP focal person   Other (Specify)____________________ | | | | | |  |
| 3.2 | How was the information communicated to you? | 1. Through lane meeting 2. Through HH visit   Other (specify)____________________ | | | | | |  |
| 3.3 | How frequently do you receive the information? | 1. Once a month 2. Once after 2-3 month   Other (Specify)____________________ | | | | | |  |
| 4 | Do you know the correct dose of Wawamum? | 1 =Yes [Once daily or 30 per month]  2 = No | | | | | | 99 = NA |
| 5 | Do you know the correct dose of MNP? | 1 =Yes [On alternate day or 15 per month] 2 = No | | | | | | 99 = NA |
| 6 | Do you know the correct dose of WSB? | 1 =Yes [Once daily or 2 packets per month] 2 = No | | | | | | 99 = NA |
| 7 | How do you use Wawamum?  (Method) | 1. As it is without mixing up anything 2. By mixing up with milk 3. By mixing up with water   Other specify ………………………….. | | | | | | 99 = NA |
| 8 | How do you use MNP?  (Method) | 1. By mixing up with semi solid or solid foods 2. By mixing up with liquids   Other specify ……………………….. | | | | | | 99 = NA |
| 9 | How do you use WSB?  (Method) | 1. By backing bread/roti 2. By preparing sweet/Halwa   Other specify ……………………….. | | | | | | 99 = NA |
| 10 | Whom do you feed **Wawamum?** | 1. Children 6-23 months  2. Children 24-59 months  3. Other family members  Other (Specify): ___________________ | | | | | | 99 = NA |
| 10.1 | If sharing please specify the reasons of sharing for **Wawamum**? |  | | | | | | 99 = NA |
| 11 | Whom do you feed **MNP?** | 1. Children 6-23 months  2. Children 24-59 months  3. Other family members  Other (Specify): __________________________ | | | | | | 99 = NA |
| 11.1 | If sharing please specify the reasons of sharing for **MNP**? |  | | | | | | 99 = NA |
| 12 | Whom do you feed **WSB?** | 1. PW  2. LM  3. Other family members  Other (Specify): __________________________ | | | | | | 99 = NA |
| 12.1 | If sharing please specify the reasons of sharing for **WSB**? |  | | | | | | 99 = NA |
| 13 | Are you willing to use **Wawamum** in future? | 1 = Yes | 2 = No (Specify Reasons)  _______________________ | | | | | 99 = NA |
| 14 | Are you willing to use **MNP** in future? | 1 = Yes | 2 = No (Specify Reasons)  _______________________ | | | | | 99 = NA |
| 15 | Are you willing to use **WSB** in future? | 1 = Yes | 2 = No (Specify Reasons)  _______________________ | | | | | 99 = NA |
| 16 | Are you willing to purchase and use **Wawamum** in future? | 1 = Yes | 2 = No | | | | | 99 = NA |
| 17 | Are you willing to purchase and use **MNP** in future? | 1 = Yes | 2 = No | | | | | 99 = NA |
| 18 | Are you willing to purchase and use **WSB** in future? | 1 = Yes | 2 = No | | | | | 99 = NA |
| **Section B: Indicators related to IYCF** | | | | | | | | |
| 19 | What is the correct time to initiate breast feeding? | 1. Within one hour after birth 2. Within two hours after birth   Other (Specify): _______________________________ | | | | | | |
| 20 | When did you initiate breastfeeding to your last child? | 1. Within an hr after of birth 2. Within two hrs after birth 3. NA in case of first pregnancy   Other (Specify): _______________________________ | | | | | | |
| 21 | What is the recommended duration of exclusive breast feeding? | Duration in Days: _____Duration in Months: _______ | | | | | | |
| 22 | What was the duration of exclusive breast feeding for your last child? | Duration in Days: _____ Duration in Months: ______  99 = NA | | | | | | |
| 23 | What is the correct/recommended time to initiate complementary feeding in young children? | 1. After _____________ months 2. = Don’t Know | | | | | | |
| 24 | When did you start to feed weaning diet/complementary feeding to your last child? | After _____________ months 99 = NA | | | | | | |
| 25 | How frequently your LHW visits your household? | After _________ days  After _________months  Never visited ------------------------- 00 | | | | | | |
| 26 | Does the LHW perform Height for your children? | 1 = Yes | | | | | 2 = No | |
| 26.1 | If yes, how frequently she is performing? | After _________ days  After _________months | | | | | | |
| 27 | Does anyone ever visit your household for the purpose of monitoring? | 1 = Yes | | | | 2 = No | | |
| 27.1 | If yes, who visited? | 1. LHS 2. WFP team   Other (Specify): _____________________________ | | | | | | |

Name of Interviewer: _______________________ Date: _____________ Checked by: _________________
