# Supplementary material for: Food supplements to reduce stunting in Pakistan: a process evaluation of community dynamics shaping uptake
Source: BMC Public Health. 2020 Jul 2;20:1046. doi: 10.1186/s12889-020-09103-8 (PMC7331235; doi:10.1186/s12889-020-09103-8)
Supplement: Supplementary file 2 — Additional file 2: Annex 2. FGD Guide Focus Group Discussion guide used in the study [file 12889_2020_9103_MOESM2_ESM.docx]

For Mothers

Focus Group Discussion Guide

| Supplements |
| --- |
| Q.1 Have you ever been told about stunting among children?  Probes:   - Who told you about stunting - What is stunting - Why stunting happens - Importance of nutrition/supplements   Q.2: What are the benefits of supplements?  Probes:  Wawamum:   - Wawamum; How it is valuable and nutritious - What are the benefits of Wawamum - Why Wawamum is not beneficial   MNP:   - MNP; How it is valuable and nutritious - What are the benefits of MNP - Why MNP is not beneficial   WSB:   - WSB; How it is valuable and nutritious - What are the benefits of WSB - Why WSB is not beneficial   Q. 3: Who consumes these supplements?  Wawamum   - Who should consume Wawamum - Who consumes Wawamum - Is Wawamum shared in the household? - Why do share wawmum with other family members? Reasons please - Lack of usage (if it happening-reasons)   WSB:   - Who should consume WSB - Who consumes WSB - Is WSB shared in the household? - Why do share WSB with other family members? Reasons please - Lack of usage (if it happening-reasons)   MNP:   - Who should consume MNP - Who consumes MNP - Is MNP shared in the household? - Why do share MNP with other family members? Reasons please - Lack of usage (if it happening-reasons)   Q.4: What features of supplements are liked and features are less liked?  Probe:  Wawamum:   - Taste - Odour - Colour - Texture - Packaging   MNP:   - Taste - Odour - Colour - Texture - Packaging   WSB:   - Taste - Odour - Colour - Texture - Packaging   Q. 5: Information and communication?  Probe:   - From where do you receive these supplement - How do you receive information about these supplements - Who tells you about usage of these supplements - Do you have sufficient information about these supplements - How information sharing can be better communicated - Who should provide the information to you   Q. 6: Do you get these supplements regularly  Probe:   - Who receive these supplements - Do you receive these supplements regularly - Do you receive enough supplements as per required amount of usage - Do you get supplements each time you attempt to receive |

For Grand Mothers

Focus Group Discussion Guide

|  |
| --- |
| Q.1: What are the benefits of supplements?  Probes:  Wawamum:   - Wawamum; How it is valuable and nutritious - What are the benefits of Wawamum - Why Wawamum is not beneficial   MNP:   - MNP; How it is valuable and nutritious - What are the benefits of MNP - Why MNP is not beneficial   WSB:   - WSB; How it is valuable and nutritious - What are the benefits of WSB - Why WSB is not beneficial   Q. 2: Who consumes these supplements?  Wawamum   - Who should consume Wawamum - Who consumes Wawamum - Is Wawamum shared in the household? - Why do share wawmum with other family members? Reasons please - Lack of usage (if it happening-reasons)   WSB:   - Who should consume WSB - Who consumes WSB - Is WSB shared in the household? - Why do share WSB with other family members? Reasons please - Lack of usage (if it happening-reasons)   MNP:   - Who should consume MNP - Who consumes MNP - Is MNP shared in the household? - Why do share MNP with other family members? Reasons please - Lack of usage (if it happening-reasons)   Q.3: What features of supplements are liked and features are less liked?  Probe:  Wawamum:   - Taste - Odour - Colour - Texture - Packaging   MNP:   - Taste - Odour - Colour - Texture - Packaging   WSB:   - Taste - Odour - Colour - Texture - Packaging |

For SPOUSES

Focus Group Discussion Guide

|  |
| --- |
| Q.1: What are the benefits of supplements?  Probes:  Wawamum:   - Wawamum; How it is valuable and nutritious - What are the benefits of Wawamum - Why Wawamum is not beneficial   MNP:   - MNP; How it is valuable and nutritious - What are the benefits of MNP - Why MNP is not beneficial   WSB:   - WSB; How it is valuable and nutritious - What are the benefits of WSB - Why WSB is not beneficial   Q. 2: Information and communication?  Probe:   - From where do you receive these supplement - How do you receive information about these supplements - Who tells you about usage of these supplements - Do you have sufficient information about these supplements - How information sharing can be better communicated - Who should provide the information to you   Q. No: 3 Delivery point and purchasing of supplements in future   - Where from do get these supplements - Who receive these supplements - If these supplements are available in the market in the future will you purchase - At what prices ( ask separately): - Wawamum: price for one packet; - what price for one MNP sachet; - What price for WSB one pack |

For Elders

Focus Group Discussion Guide

|  |
| --- |
| Q.1: What are the benefits of supplements?  Probes:  Wawamum:   - Wawamum; How it is valuable and nutritious - What are the benefits of Wawamum - Why Wawamum is not beneficial   MNP:   - MNP; How it is valuable and nutritious - What are the benefits of MNP - Why MNP is not beneficial   WSB:   - WSB; How it is valuable and nutritious - What are the benefits of WSB - Why WSB is not beneficial   Q. No: 2. Delivery point and purchasing of supplements in future   - Where from do get these supplements - Who receive these supplements - If these supplements are available in the market in the future will you purchase - At what prices ( ask separately): - Wawamum: price for one packet; - what price for one MNP sachet;   What price for WSB one pack  Q. No 3: How can we improve such programs like this on in the future to enhance our children’s health and nutrition   - What can be your role as ELDERS - What can be role of local government - What can be role of women especially mother - What role can village based organization play - What can role local government |

LADY HEALTH WORKERs

Focus Group Discussion Guide

| Supplements |
| --- |
| Q.1: What features of supplements are liked and features are less liked?  Probe:  Wawamum:   - Taste - Odour - Colour - Texture - Packaging   MNP:   - Taste - Odour - Colour - Texture - Packaging   WSB:   - Taste - Odour - Colour - Texture - Packaging   Q No 2: In your view what are the strengths and weakness in this food supplements intervention?   - How is the coordination? Among you (LHW), WFP and DoH - Do you receive required amount of supplements regularly - Besides your regular job responsibilities how you manage this role - Have you ever failed to perform your due role in the project? How? Why it happens/happened |
