# Supplementary material for: Food supplements to reduce stunting in Pakistan: a process evaluation of community dynamics shaping uptake
Source: BMC Public Health. 2020 Jul 2;20:1046. doi: 10.1186/s12889-020-09103-8 (PMC7331235; doi:10.1186/s12889-020-09103-8)
Supplement: Supplementary file 3 — Additional file 3: Annex 3. KII Guide Key Informant Interview guide used in the study [file 12889_2020_9103_MOESM3_ESM.docx]

Effectiveness of food based interventions to prevent stunting among children in Thatta & Sajawal Districts, Sindh Province

End-line Process Assessment

Tool for Key Informant Interviews

Name of Taluka: ___________________________ Name of UC: ___________________________

Name of Village/Area: ___________________________________________________________________

| Background Information: | |
| --- | --- |
| 1. Name: _________________ 2. Age: _______ 3. Education: _______ 4. Occupation: _____________ | |
| 1 | Food supplementation |
| 1.1 | Food delivery system |
|  | Do you think that the measures that were adopted for delivery of food based supplements were appropriate and effective? |
|  | Can you, please throw light on overall implementation process; please tell us the weakness and strengths?  Probes:   - District distribution system - Capacity of front line workers i.e. LHW - Supply chain - Storage of food - How to improve program operations |
| 1.2 | End user barriers |
|  | In your opinion was this food based supplement program for <5 years children and pregnant & lactating mother beneficial for the community in Thatta/Sujawal?  Probes:   - Why yes/why no - Acceptability for food supplements - Cultural/religious beliefs |
|  |  |
|  | How could the acceptance of food based supplement program be enhanced in the community?  Probes:   - Measures to enhance uptake |
| 1.3 | Unintended consequences |
|  | In your opinion what were the consequences of food supplement program, if any, encountered during the program? (Negative or positive)  Probes:   - Dependency on supplement food - Crowding out of local food items - Demand overtaking supply - Pilferage - Better linkages with LHW - Women empowerment - Crossing over from control group - Political capture |
| 2 | Preventive care |
|  | What is your opinion on current preventive care provided in your area? ( ONLY for UC Chairman)  Probes:   - Type of provider - Scope of services - Frequency/interaction |
| 3 | Information & communication |
|  | In your opinion what are the various approaches for information sharing and communication adopted by the LHWs  Probes:   - Nature of communication - Type of information |

Name of Interviewer: ________________________ Date of Interview: _______________________

Name of Team Leader: __________________Date of form checked by TL: ______________
